
JOURNAL INFORMATION
==============================
NLM Title Abbreviation: Innov Surg Sci
Journal ID: Innov Surg Sci
NLM Journal ID: 101708165
Journal ID: iss

Innovative Surgical Sciences

EISSN: 2364-7485
Publisher: De Gruyter

ARTICLE INFORMATION
==============================
PMCID: PMC11917183
PMID: 40110099
DOI: 10.1515/iss-2025-2001
Article ID: iss-2025-2001
Article version: 1
Subjects: Article

Abstracts interdisciplinary

Electronic publication date: 2025 Mar 19
Publication date: 2025 Mar
Volume: 10
Issue: Suppl1
First page: 1
Last page: 39
Copyright: © 2025 by Walter de Gruyter Berlin/Boston
Copyright year: 2025
Copyright holder: De Gruyter
License: This work is licensed under the Creative Commons Attribution 4.0 International License.
License URL: https://creativecommons.org/licenses/by/4.0/

Page count: 39

==============================

Abstracts – DCK 2025 – 142nd Congress of the German Society of Surgery – Munich, March 26–28 • DOI 10.1515/iss-2025-2001
Innov Surg Sci 2025; 10 (Special Suppl 1): s1–s39

### Enhancing patient comprehension in liver resection informed consent: The EPIC-AR Trial – A Randomized Controlled Trial using augmented Reality and 3D Visualization Technologies

ID: 673

**Kategorie: interdisziplinäre Chirurgie - AI und AR in der operativen Planung und Durchführung**

Schaima Abdelhadi[1], Flavius Sandra-Petrescu[1], Christoph Reissfelder[1], Cui Yang[1]

[1]*Universitätsmedizin Mannheim, Chirurgische Klinik, Mannheim, Deutschland*

**Background:**
Informed consent is a fundamental ethical requirement and a legal obligation in all surgical procedures. It involves a clear, mutual understanding between the patient and the surgeon about the nature of the surgery, potential risks, benefits, and alternatives. However, the complexity of surgical procedures, especially liver resection, poses significant challenges to the informed consent process. Traditional methods, predominantly verbal explanations supplemented by static images, often fall short in adequately conveying the intricate details of the surgery. This gap in understanding can lead to anxiety, dissatisfaction, and a lack of informed decision-making among patients. Previous research has demonstrated that three-dimensional (3D) presentations improve the identification of liver anatomy and 3D liver model-based surgical education improves preoperative decision-making and patient satisfaction. Recent advancements in augmented reality (AR) and 3D visualization technologies offer a more dynamic and interactive way of presenting complex surgical information, potentially enhancing patient understanding and satisfaction. However, the effectiveness of these technologies in the context of informed consent for liver resection remains underexplored. The primary objective of this trial, the EPIC-AR Trial (Enhancing Patient Comprehension in Liver Resection Informed Consent using Augmented Reality and 3D Visualization Technologies), is to assess whether the use of AR and 3D visualization technologies can improve the clarity and effectiveness of informed consent dialogues compared to traditional methods.

**Materials and methods:**
This is a prospective randomized, controlled trial with two parallel study groups. The primary objective of this trial is to evaluate the impact of augmented reality (AR) and 3D visualization technologies on patient satisfaction during the informed consent process for liver resection.
The following hypothesis will be tested:
H0: Both interventions are equivalent.
H1: via the usage of AR and 3D visualization technologies during surgical consent, patients get informed more
comprehensibly, feel safer und less anxious about their upcoming surgery and feel treated by state-of-the-art
information technology.
Patients scheduled for elective LR will be screened for inclusion in the trial. Patients meeting the eligibility criteria will be enrolled into the study. Written informed consent is obtained at least one day before surgery.

**Results:**
Trial Duration and Dates:
Start to end dates: **February 2025 to January 2025**
Statistical analysis and report: **February 2025**

**Conclusion:**
**Trial objectives and endpoints**
**<u>Primary objective</u>**
The primary objective of the EPIC-AR Trial is to evaluate the impact of augmented reality (AR) and 3D visualization technologies on patient satisfaction during the informed consent process for liver resection. This will be assessed using detailed patient satisfaction surveys immediately following the consent dialogue.
**<u>Secondary objectives</u>**
**Patient Comprehension:** To assess the accuracy of patient understanding of the surgical procedure, risks, benefits, and alternatives, as measured through structured comprehension tests conducted post-consent dialogue

Open Access. © 2025 The Author(s), published by De Gruyter. This work is licensed under the Creative Commons Attribution 4.0 International License.

**Anxiety Assessment:** To compare pre and post-consent anxiety levels in patients, using validated anxiety scales, to determine if the AR and 3D visualization approach reduces patient anxiety more effectively than traditional methods.
**Efficiency of Consent Process:** To analyze the time efficiency of the informed consent process with and without the use of AR and 3D visualization technologies.
**Assessment of endpoints**
**Patient satisfactory:** Patient satisfaction represents the primary endpoint of the EPIC-AR trial. It will be assessed immediately after the consent process by a questionnaire specifically developed for this study and postoperative by the EORTC Questionnaire (IN-PATSAT32).
**Patient comprehension:** Patient knowledge and understanding of the own disease and the surgery including the surgical approach, risks and benefits will be assessed by a questionnaire specifically developed for this study. The comprehension assessment will be conducted before and immediately after the consent dialogue, as well as at the postoperative visit.
**Patient anxiety levels:** Patient anxiety levels will be evaluated by using the Visual Analog Scale (VAS, 1–10) and the State-Trait-Anxiety-Inventory, consisting of the State-Anxiety-Score (STAI) and the Trait-Anxiety-Score (TAI). In this study we will use the German short version of the STAI, including 10 statements for the state anxiety and 10 statements for the Trait anxiety of the patient. The anxiety level assessment will be conducted before and immediately after the consent dialogue, as well as at the postoperative visit.
**Time Efficiency:** The duration of each consent dialogue will be recorded and analyzed for time efficiency.

### Evaluation of the MS Teams application via mixed reality in the context of visceral medicine
ID: 882

**Kategorie: interdisziplinäre Chirurgie - AI und AR in der operativen Planung und Durchführung**

Daniela Salzmann[1], Navid Tabriz[2], Verena Müller[2], Marc-Angelo Bisotti[1], Verena Uslar[2], Sirko Pelzl[1], Dirk Weyhe[2]

[1]*apoQlar GmbH, Hamburg*, [2]*PIUS-Hospital, Allgemein- und Viszeralmedizin, Oldenburg*

**Background:**
In clinical applications, Remote Assist systems have been used for more than 20 years for telepresence or telementoring in various medical fields such as urology, general surgery, neurosurgery, etc. Remote Assist can be used within a hospital, outside the hospital or across national borders. Surgical experts and mentors are connected e.g. via a computer, video conferencing system, workstation or increasingly via augmented reality/mixed reality glasses. A meta-analysis by Erridge et al. from 2019 showed that telementoring systems have a similar safety and benefit profile to support from an on-site mentor. One solution that offers remote assistance via mixed reality is VSI HoloMedicine. The aim of the qualitative, explorative study is to evaluate the Teams MR application of VSI HoloMedicine® in the context of visceral medicine in various scenarios in everyday clinical practice.

**Materials and methods:**
As part of the study, an expert is connected via remote assist using MS Teams of the VSI HoloMedicine® software. The attending physician wears the MR glasses and uses them to share his field of vision and sound. During the trial, the connected expert supports his colleague in diagnosis and treatment, e.g. on-call service, emergency department or in the operating theater. Individual semi-structured interviews are then conducted with the connected expert and the attending physician. The focus is on the following aspects: Performance of the MS Teams integration (remote support, 3D hologram, streaming and voice quality), virtual collaboration, evaluation of user-friendliness, advantages and disadvantages as well as user satisfaction. Finally, a survey is conducted on demographic and job-related aspects as well as the mental workload (NASA-TLX) during use. The testing and interviews were recorded and transcribed. The interviews are evaluated using qualitative content analysis on the basis of an inductive-deductive category system.

**Results:**
The study is ongoing at the PIUS Hospital in Oldenburg. 11 patient cases and 22 interviews have already been conducted. Initial results show that very good communication and image transmission in real time over spatial distances (e.g. Thailand) is possible. The light-dark regulation is limited during the transmission of an ultrasound field of view. This should therefore also be transmitted separately directly from the ultrasound device. Suggestions for improvement include enlarging the field of view or integrating loudspeakers into the HoloLens. The application is perceived as simple and self-explanatory, satisfaction is rated as good and its use is recommended to colleagues.

**Conclusion:**
The study presents the gather initial experience on the use of the VSI-HoloMedicine Remote Assist function in visceral medicine. The application allows virtual support of the diagnostic and treatment process in visceral medicine from a distance. The Remote Assist function offers a potential solution to enhance quality, efficiency, access to care, and patient safety. The performance of the VSI-HoloMedicine Remote Assist function and the safety of the application have been assured. However, limitations of the study include a small sample size and limited transferability of results to other hospitals. Further research should base on randomized controlled trials to demonstrate clinical benefit, efficacy, and quality of care of the application.

### Palliative Plastic-Reconstructive Surgery in Interdisciplinary Structures

ID: 241

**Kategorie: interdisziplinäre Chirurgie - End-of-life surgery**

Sophia Diesch[1] [1], Vanessa Brebant[1], Silvan Klein[1], Lukas Prantl[1]

[1]*Universitätsklinikum Regensburg, Abteilung für Plastische, Hand- und Wiederherstellungschirurgie, Regensburg, Deutschland*

**Background:**
Due to the improvement in treatment options for severe tumor diseases and the development of new oncological therapies, the survival time of patients with a palliative prognosis is increasing. However, palliative patients often suffer from symptoms that severely limit their quality of life. In clinical practice, there is currently little interface between palliative medicine and plastic-reconstructive surgery, although small reconstructive interventions could significantly alleviate symptoms and improve quality of life. An interdisciplinary palliative conference could greatly optimize the treatment of these patients.

**Materials and methods:**
A review will demonstrate the value of an interdisciplinary palliative conference and the role of plastic surgery in the palliative setting. Additionally, case studies from our institution will be used to present established treatment algorithms.

**Results:**
Stage-appropriate surgical procedures can improve the quality of life for palliative patients suffering from symptoms such as recurrent infections, severe wounds, impending bleeding, social isolation, and psychological distress. The current literature contains only a few significant studies on surgical approaches, making treatment decisions largely case-based.
The introduction of an interdisciplinary palliative conference allows for early optimization of patient treatment, including potential surgical interventions. A thorough cost-benefit analysis should be conducted for surgical options, considering both free and local flap techniques as potential therapies.

**Conclusion:**
In the context of an interdisciplinary palliative conference, patient cases should be discussed regularly to treat symptoms that significantly impact quality of life as early as possible. The treatment should be purely symptomatic, with the goal of improving quality of life rather than prolonging survival. The institution's established treatment algorithm serves as a decision-making aid for plastic-reconstructive approaches in the palliative setting.

Abstracts – DCK 2025 – 142nd Congress of the German Society of Surgery – Munich, March 26–28 • DOI 10.1515/iss-2025-2001
Innov Surg Sci 2025; 10 (Special Suppl 1): s1–s39

**Palliative decompression for malignant bowel obstruction after esophagectomy with colon interposition – The alternative when standard decompression methods are not an option.**
ID: 745

**Kategorie: interdisziplinäre Chirurgie - End-of-life surgery**

Ahmed Alwali[1], Kristina Bath[2], Imad Kamaleddine[1], Clemens Schafmayer[1]

[1]*Klinik für Allgemeine Chirurgie, Thorax, Gefäß- und Transplantationschirurgie - Universitätsmedizin Rostock, Rostock, Deutschland*, [2]*Institut für Diagnostische und Interventionelle Radiologie, Kinder- und Neuroradiologie, Rostock, Deutschland*

**Background:**
Decompression of the gastrointestinal tract in patients with malignant bowel obstruction offers symptomatic relief in palliative care, particularly in cases with complex abdominal conditions. Standard methods, such as percutaneous endoscopic gastrostomy (PEG) or jejunostomy, typically work well but are not viable in certain cases due to factors like peritoneal carcinomatosis or postoperative alterations in gastrointestinal anatomy. In such cases, temporary relief is often provided by nasogastric tubes, which are not suitable for long-term use in these challenging situations. Alternatives, such as transesophageal gastrostomy tubes[1] and percutaneous transhepatic duodenostomy[2], have been reported. Additionally, percutaneous feeding gastrostomy in patients with partial gastrectomy via a transhepatic approach, guided by CT, has been successfully reported with no significant complications.[3] This report details an innovative approach to decompression in a patient with peritoneal metastases following esophagectomy with colon interposition, who presented with recurrent vomiting due to malignant bowel obstruction and a "frozen abdomen." The procedure involved a successful transhepatic decompression performed under CT guidance and with endoscopic assistance.

**Materials and methods:**
After an unsuccessful attempt at endoscopic decompression due to the absence of diaphanoscopy, caused by the adhesion of the liver's left lobe to the colon interposition, we opted for percutaneous transhepatic decompression of the colon interposition. The procedure involved puncturing the colon interposition using a 22-gauge percutaneous transhepatic needle through the left lobe of the liver, guided by computed tomography (CT) and real-time visualization by using endoscopy. Following the puncture, tract dilation over a guidewire was performed, and 15-French balloon PEG-catheter was placed using the Seldinger technique.

**Results:**
The procedure was completed without any immediate complications. Four hours post-procedure, a control ultrasound showed no signs of bleeding. The decompression catheter remained in place, and at a 1-month follow-up, the patient reported a significant reduction in vomiting symptoms, and no major complications were observed.

**Conclusion:**
This report highlights that percutaneous transhepatic decompression, guided by CT and endoscopy, can serve as an alternative to traditional palliative decompressive techniques, particularly when anatomical changes prevent the use of standard methods.

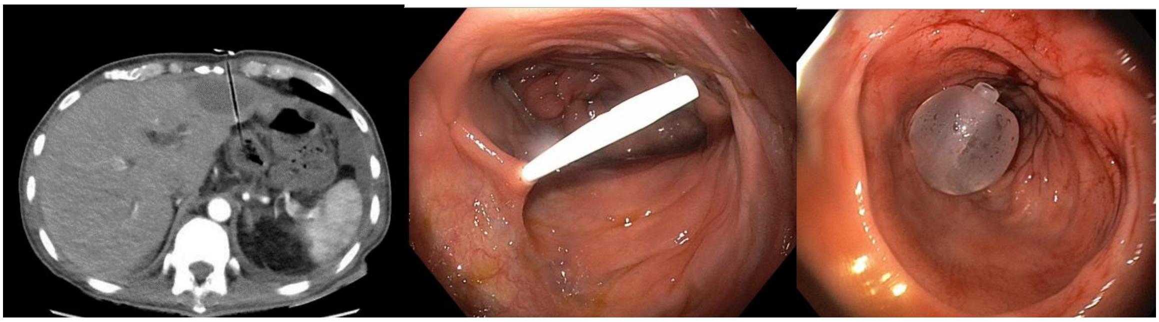

References

*1. Zhu C, Platoff R, Ghobrial G, Saddemi J, Evangelisti T, Bucher E, Saracco B, Adams A, Kripalani S, Atabek U, Spitz FR, Hong YK. What to do When Decompressive Gastrostomies and Jejunostomies are not Options? A Scoping Review of Transesophageal Gastrostomy Tubes for Advanced Malignancies. Ann Surg Oncol. 2022 Jan;29(1):262-271. doi: 10.1245/s10434-021-10667-x. Epub 2021 Sep 21. PMID: 34546480.*

*2. Moriwaki Y, Otani J, Sawada Y, Okuda J, Niwano T, Ntta T, Ohshima C. Percutaneous transhepatic duodenostomy for a gastrectomy case with CT guidance and real-time visualization by an ultrasound and endoscopy. Nutrition. 2015 Sep;31(9):1168-72. doi: 10.1016/j.nut.2015.04.004. Epub 2015 May 7. PMID: 26233876.*

*3. Kanazawa S, Naomoto Y, Hiraki Y, Yasui K, Matsuno T. Percutaneous feeding gastrostomy in patients with a partial gastrectomy: transhepatic approach with CT guidance. Abdom Imaging. 1995 Jul-Aug;20(4):302-6. doi: 10.1007/BF00203358. PMID: 7549730.*

### PIPAC – more than a palliative treatment in selected patients?

ID: 924

**Kategorie: interdisziplinäre Chirurgie - End-of-life surgery**

Esther Anna Biesel[1], Gabriel Seifert[1], Stefan Fichtner-Feigl[1], Hannes P. Neeff[1]

[1]*Universitätsklinikum Freiburg, Abteilung für Allgemein- und Viszeralchirurgie, Freiburg, Deutschland*

**Background:**
Pressurized intraperitoneal aerosol chemotherapy (PIPAC) is an innovative and minimally invasive treatment approach for patients with peritoneal metastases, particularly those arising from colorectal, gastric and ovarian cancers, as well as for primary peritoneal malignancies. Initially developed as a palliative treatment modality accompanying systemic chemotherapeutic treatment, PIPAC therapy shows encouraging results also in long-term treatment or in a neoadjuvant setting in selected cases.

**Materials and methods:**
The PIPAC program at our institution started in October 2018. All patients scheduled for PIPAC therapy were enrolled in a prospectively maintained SPSS database. Variables provide information on baseline characteristics of patients, access rate and details on PIPAC therapy as well as on oncological follow-up. We identified two patients that exceeded the initially planned three repetitive PIPAC procedures due to promising treatment responses, so that the PIPAC procedure was performed as neoadjuvant treatment and as long-term treatment, respectively.

**Results:**
From October 2018 until September 2024, we performed altogether 98 PIPAC procedures in 46 patients with a primary access rate of 74.2%. The most frequent primary tumors were colorectal carcinomas and gastric cancer. Our first case is a male patient, aged 68, with peritoneal mesothelioma, firstly diagnosed in June 2018. After open surgical exploration, which revealed an irresectable tumor burden, the patient was scheduled for systemic chemotherapy which was stopped due to intolerable adverse effects. Instead, he received 11 repetitive PIPAC procedures (Cisplatin/Doxorubicin) with satisfying tumor control. When developing progressive disease in November 2021, immunotherapy was started which was well tolerated. After 32 cycles (29 months) of immunotherapy and again signs of progressive disease, the patient was rescheduled for further PIPAC therapies and is still alive and in good medical condition. The second case is another male patient, aged 64, with synchronous peritoneal metastases of a gastric cancer, who was initially under systemic chemotherapeutic treatment. He received PIPAC treatment (Cisplatin/Doxorubicin) under palliative intent. After excellent tumor response at re-staging, subtotal gastrectomy was performed after a total of three PIPAC procedures and revealed an ypT4ypN3a-tumor with only minimal regression in the primary tumor, but subtotal regression in peritoneal metastases, so that PIPAC therapy was continued, leading to an overall survival of 30 months with good quality of life.

**Conclusion:**
In spite of being introduced as a palliative treatment in order to alleviate symptoms of peritoneal metastases, especially malignant ascites, PIPAC therapy seems to represent a promising therapeutic tool in selected patients as a long-term therapy or in a neoadjuvant setting. Excellent tumor response after systemic therapy or long-standing stable disease warrant the repeated use of PIPAC in selected patients. The role of PIPAC therapy in a distinct “a priori” neoadjuvant setting still remains unclear.

### Complex Reconstruction of the Distal Radius in Recurrent Giant Cell Tumor: Case Report and Long-Term Outcomes

ID: 610

**Kategorie: interdisziplinäre Chirurgie - Extremitätenrekonstruktion**

Judith Döringer[1], Esther Vögelin[1], Dominique Merky[1]

[1]*Inselspital, Bern, Schweiz*

**Background:**
Giant cell tumors of the bone present a significant challenge and often require complex reconstructive procedures, particularly in the upper extremity, to preserve both limb integrity and functionality.

**Materials and methods:**
We present the case of a 28-year-old female patient diagnosed with a biopsy-confirmed giant cell tumor of the right dominant distal radius in May 2020. The initial treatment involved intralesional curettage, Argon beam therapy, defect filling with PMMA, and combined osteosynthesis. After a favorable initial outcome, the patient reported a new, tender swelling on the dorsal aspect of the distal radius nearly one year postoperatively, which was diagnosed as a recurrence. In response, a year-long systemic therapy with Denosumab was initiated, stabilizing the tumor. However, the development of radiocarpal and distal radioulnar joint (DRUJ) arthritis, compounded by partially intra-articular cement placement, led to significant pain and functional impairment, making the patient unable to work and prompting her to opt for a second surgery. Thus, two years postoperatively, in June 2022, after interdisciplinary consultation and 3D planning, reconstruction of the right distal radius was performed using a free, microvascular osteocutaneous fibula flap from the left leg, applied in a double-barrel technique with simultaneous RSL fusion. The sigmoid notch was reconstructed using an ipsilateral Brachioradialis tendon strip, while the Palmaris Longus tendon was used to repair the TFCC and extensor retinaculum.

**Results:**
Histological examination revealed clear bony and soft tissue resection margins. Seven months postoperatively, full consolidation of the fibula graft proximally and at the fibulo-lunate site was observed, with partial consolidation at the fibulo-scaphoid site. The patient's pain decreased rapidly and the range of motion and strength in her right wrist improved. At the most recent follow-up in May 2024, the patient expressed high satisfaction and reported no substantial limitations in her daily activities or professional life. There were no signs of local recurrence or lung metastases.

**Conclusion:**
Through complex bone and soft tissue reconstruction, this young patient’s destructive giant cell tumor of the right dominant radius was successfully treated curatively, despite recurrence, restoring a functional, pain-free limb.

### Arthroscopic versus open surgery for scaphoid pseudarthrosis: A retrospective case-control study

ID: 617

**Kategorie: interdisziplinäre Chirurgie - Extremitätenrekonstruktion**

Judith Döringer[1], Esther Vögelin[1], Dominique Merky[1], Remy Liechti[1], Lena Dietrich[1]

[1]*Inselspital , Bern, Schweiz*

**Background:**
Currently, there is no consensus on the optimal surgical technique for treating scaphoid pseudarthrosis—whether arthroscopic or open surgery is preferable. Open bone grafting may provide better carpal alignment compared to arthroscopic methods, but the clinical significance of this difference remains unclear. The aim of this retrospective study is to compare the clinical and radiological outcomes of arthroscopic versus open surgery for scaphoid pseudarthrosis.

**Materials and methods:**
We conducted a retrospective case-control study, evaluating patients who underwent surgery for scaphoid pseudarthrosis between October 2015 and March 2023. Inclusion criteria required patients to be over 18 years old, with no preexisting degenerative disease or significant concomitant injuries that would hinder standardized follow-up treatment. The outcomes compared between the two groups included wrist range of motion, grip strength, complication rates and reoperation rates. Radiological data focused on bone consolidation, incidence of post-traumatic wrist osteoarthritis and changes in the length of the scaphoid axis.

**Results:**
Arthroscopic surgery appears to be a viable alternative to the open approach, offering similar postoperative outcomes in terms of pain, range of motion and grip strength. The shorter surgical time associated with arthroscopy is a potential advantage, though it should be noted that there is an initial learning curve. Given the potential for cost savings and the feasibility of performing arthroscopic procedures on an outpatient basis, this technique may become increasingly favored.

**Conclusion:**
This study supports the use of an arthroscopic approach for treating scaphoid pseudarthrosis. It offers a time-efficient, cost-effective alternative to the open approach, with no compromise in function and faster time to bone consolidation.

**Best management of mesenteric ischemia: a series of two cases**
ID: 394

**Kategorie: interdisziplinäre Chirurgie - How I do it – Mesenteriale Ischämie**

Andreea Sandica[1], Mohammed Abdulla[1], Iurii Snopok[1], Anushe Weber[2], Andreas Schnitzbauer[1], Richard Viebahn[1]

[1]*Universitätsklinikum Knappschaftskrankenhaus Bochum, Chirurgie , Bochum, Deutschland*, [2]*Universitätsklinikum Knappschaftskrankenhaus Bochum, Radiologie , Bochum, Deutschland*

**Background:**
Mesenteric ischemia is an uncommon disease, especially in young patients, with a pontentially lethal outcome. This is even more so if the condition is not properly diagnosed or treated immediately. We present a series of cases which presented with this disease at our centre and try to analize if the different outcomes could be unified by a certain course of treatment to increase the probability of surviving this lethal condition.

**Materials and methods:**
Case series

**Results:**
**Case 1**
A 48-year-old male presented to the emergency room with progredient abdominal pain, nausea and diarrhea. Pain symptoms had been exacerbating over the day of presentation in the emergency room. Upon clinical examination, the patient presented with a distended abdomen and diffuse abdominal pain. Laboratory results showed slightly elevated infection parameters and a high Troponin T serum level. Further anamnesis revealed thrombosis of the pulmonary artery in the history of the patient, under current anticoagulation with Apixaban. A CT scan of the abdomen with CT angiography showed an extended thrombotic occlusion of the SMA with signs of ischemic lesions of the bowel. The patient was immediately taken to radiology for interventional angiography. Due to the strongly consolidated thrombotic material in the SMA, it was only possible to reperfuse the proximal arcades of the mesenteric circulation. After this, the patient was taken for diagnostic laparoscopy. Intraoperatively, the terminal ileum showed progressive signs of ischemia and was resected over a lenght of 20 cm. The patient was postoperatively transfered to the ICU for sepsis therapy. Upon second look the following day, almost the entire small bowel including the coecum showed extensive signs of ischemia and was resected with 120 cm of duodenum remainig vital. Due to further intestine swelling and peritonitis, the abdomen was left open for vacuum therapy and closed upon a further third intervention. Upon further treatment with simprovement of septic values, the patient was able to be extubated and was moved from the ICU. He developed a short bowel syndrome, which ameliorated during further hospitalisation with no further complications and was able to be dismissed from stationary care. He was given the contacts to a nutrition centre for further help and of a haematological centre for further diagnostic.
**Case 2**
A 69-year-old patient was transferred to our centre with exacerbated abdominal pain after gastroscopy. A CT scan was performend in the outward centre showing a celiacal stenosis/ stenosis of the superior mesenterical artery with poststenotic reperfusion. Because of paralytic ileus and elevated sepsis parameters, she was treated with prokinetic medication and antibiotics. Laboratory results also showed an elevated serum lactate level. Symptoms were regredient at first, however after two days the patient presented again with exacerbated pain, paralytic bowel and further elevation of serum lactate. She was transferred to our centre and underwent immediate laparotomy. Upon procedure, the patient showed signs of generalized peritonitis with ischemic, oedematous bowl and perforated gall bladder. Concequencially, 80 cm of small bowl were resected and cholecystectomy was performed. After the procedure, angiography was unsuccesfully performed trying to reperfuse the remaining bowl. Upon second look, further resection of small bowl and colon with jejunotransversostomy was performed because of the extent of the ischemia. The patient was left with the need for parenteral nurturing for lifetime. After being released from the ICU, she experienced worsening of the respiratory index due to pleural effusion. Thus, the pleural effusion in the left hemithorax was drained. A couple of days later, she developed an insufficiency of the intestinal anastomosis with sepsis. The patient was taken for a re-laparotomy, Here, we observed a poor blood perfusion of the liver in addition to the ischemic bowel. Due to the poor prognosis, further treatment was stopped in agreement with the patient's wishes.

**Conclusion:**

Mesenterial ischemia is a highly lethal condition at every age. Not at least because of the comparably rare incidence, the diagnosis and choise of the best course of treatment is not always easy. By presenting the two cases above, we underline however the importance of early diagnosis and treatment to prevent extended ischemic damage. A primary recanalisation of the thrombotic vessels, if successfull, is crucial for the outcome of the patient. Today, this is mostly done endovasculary, with the procedures showing continuously improving results. Abdominal compartment syndrome after recanalisation is furthermore an important factor which can limit the prognosis of these patients. This often makes decopression laparotomy and sometimes bowl resection necessary to save the lives of these patients and should also be carefully and early taken into consideration.

### Near-infrared indocyanine green angiography in recognizing bowel ischemia in emergency surgery: game changer or overrated?

ID: 733

**Kategorie: interdisziplinäre Chirurgie - How I do it – Mesenteriale Ischämie**

Anastasia Christofi[1], Thilo Traska[1], Dimitrios Dimitroulis[2]

[1]*Agaplesion Bethesda Krankenhaus Wuppertal, Allgemein-, Viszeral- und Gefäßchirurgie, Wuppertal, Deutschland*, [2]*Nationale und Kapodistrias-Universität Athen, Hellenic Minimally Invasive and Robotic Surgery (MIRS) Study Group, Athen, Griechenland*

**Background:**
Assessing bowel perfusion in emergency intestinal surgery can prove challenging even for experienced surgeons. The necessity of a technological tool assisting clinicians is undisputed. Near-infrared Indocyanine green (NIR-ICG) angiography has been increasingly used in elective colorectal surgery for the evaluation of intestinal perfusion with promising results. This review aims to answer whether a similar outcome can be observed in acute cases of bowel ischemia.

**Materials and methods:**
We conducted online research of the literature using keywords such as “indocyanine green”, “bowel”, “emergency” and “ischemia”, to identify articles concerning the use of ICG-angiography in evaluating bowel perfusion during emergency operations. PubMed was the primary database.

**Results:**
11 articles were included in this review with a total of 358 patients. Most papers showed a positive effect after using NIR-ICG-angiography, whereas one study indicated the limitations of the method by exhibiting increased reoperation and mortality rates. Moreover, a significant variation in indocyanine green (ICG) dose and fluorescence identification systems was observed.

**Conclusion:**
NIR-ICG-angiography has the potential to become a fundamental tool in emergency intestinal operations. Nevertheless, additional research, especially high-quality, randomized studies, as well as quantification techniques are still needed to support these preliminary observations.

### Delayed acute abdomen presents with mesenteric injury

ID: 928

**Kategorie: interdisziplinäre Chirurgie - How I do it – Mesenteriale Ischämie**

Jan Philipp Ramspott[1 2], Alexander Bungert[2], Gaby Kass Abd Alahad[1], Firas Diab[1], Ricarda Siems[1], Reiner Schürmann[1]

[1]*Department for General and Visceral Surgery, University Hospital Muenster, Marienhospital Steinfurt, Steinfurt, Deutschland*, [2]*Department for General, Visceral and Transplant Surgery, University Hospital Muenster, Muenster, Deutschland*

**Background:**
A 28-year-old patient was transferred to our emergency department after a car crash accident. The patient was awake and had unremarkable vital signs. He reported fastened seat belts and activated airbags. Furthermore, he reported that he left the car by himself. He neither had any comorbidities nor took any medication.

**Materials and methods:**
The patient had pain in both shoulders, in the right hemithorax, and right arm. On physical examination the abdomen was soft but showed seatbelt bruises. Blood analysis revealed leukocytosis of 20000/µl (reference 3900-10900/µl) and elevated creatine kinase of 462 U/l (reference < 190 U/l). A computed tomography scan was performed which detected small amount of ascites. No internal organ was injured but the left ulna was fractured.

**Results:**
The patient went to the intensive care unit for observation and a cast was placed on the ulna fracture. Two days after hospital admission the patient presented with an acute abdomen. We performed a new computed tomography scan and detected a larger amount of ascites compared with the initial scan. Laparoscopic exploration was undertaken but had to be switched to laparotomy due to high intraabdominal pressure. Exploration revealed a mesenteric disruption (25 cm long) of the terminal ileum without any bleeding signs. We performed an ileocecal resection and constructed an ileoascendostomy. On the 10th postoperative day, the patient was discharged home.

**Conclusion:**
Blunt abdominal trauma can be associated with severe internal organ injury (liver, kidney), whereas mesenteric injury is rare. Often, the diagnosis of mesenteric injury is prolonged due to delayed development of peritonitis. High morbidity can only be reduced by early diagnosis and the following surgical intervention.

### A multidisciplinary approach to skull base surgery

ID: 463

**Kategorie: interdisziplinäre Chirurgie - Interdisziplinäre Chirurgie der Orbita und Schädelbasis**

Peter M. Vogt[1], Khaled Dastagir[1], Joachim K. Krauss[2], Thomas Lenarz[3]

[1]*Medizinische Hochschule Hannover, Klinik für Plastische, Ästhetische, Hand- und Wiederherstellungschirurgie, Hannover, Deutschland*, [2]*Medizinische Hochschule Hannover, Neurochirurgische Klinik, Hannover, Deutschland*, [3]*Medizinische Hochschule Hannover, Klinik für Hals-, Nasen- und Ohrenheilkunde, , Hannover, Deutschland*

**Background:**
Skull base surgery involves complex surgical procedures in an anatomical area of critically exposed structures such as brain, cranial nerves, major blood vessels and the middle and inner ear.
Reconstruction aims at restoring the integrity of the skull base and protecting the underlying brain tissue against infection and other causes.

**Materials and methods:**
In a multi-disciplinary approach of neurosurgery, otorhinolaryngology, and maxillofacial surgery, the specialty of plastic surgery adds to the necessary solutions in reconstruction of extended and complex defects which cannot be handled by local or regional tissue. Skin and soft tissue defects following trauma and oncologic resections are the source of very critical defects, as ascending infections, especially on the skull base with exposed dura reconstructions, may be a risk for patients. Adequate and safe reconstruction is mandatory for prevention of infection and protection of the critically exposed regions.

**Results:**
In our interdisciplinary approach well vascularized free flaps enabling the closure of dead space are an important tool for the prevention or definitive treatment of complications.
The common problem of the “avascular neck” can be overcome by the use of arteriovenous loops or long vein grafts. The versatility of the free latissimus flap makes this tissue the most useful reconstructive choice through its features as either a simple muscle or myocutaneous flap, or chimeric tissue even for the reconstruction of very large three-dimensional defects including anatomical cavities. Other special options involve the use of free omentum grafts.

**Conclusion:**
In skull base surgery plastic surgery adds to the necessary solutions in reconstruction of extended and complex defects. The reconstructive approach is highly individualized depending on the local condition, patient needs and prognosis of the disease.

**Assessing the pre- and postoperative cranial shape of 42 infants with scaphocephaly using 3-dimensional head scans – what's the difference?**
ID: 62

**Kategorie: interdisziplinäre Chirurgie - Interdisziplinäre Chirurgie von kindlichen Fehlbildungen des Gesichts- und Gehirnschädels**

Eric Waltersbacher[1], Laura Müller[1], Christian Kruppa[1], Carola Hörz[1], Alexandra Wilke[1], Guido Fitze[1], Christoph von Schrottenberg[1]

[1]*Universitätsklinikum Carl Gustav Carus Dresden, Klinik und Poliklinik für Kinderchirurgie, Dresden, Deutschland*

**Background:**
Scaphocephaly occurs due to total or partial, premature synostosis of the cranial sagittal suture and makes 40% of all craniosynostoses, with a prevalence of 1 in 5000 infants. Indication for surgical correction in non-syndromic patients is mostly of aesthetic nature, hence assessing postoperative results may be subjective. The cephalic ratio (CR) as well as other indices describing cranial volumetric morphology (i.e. the anterior or the posterior symmetry index) can be used to objectively assess the shape of the cranial vault which makes valid comparison of postoperative results after varying surgical techniques possible.

**Materials and methods:**
Pre- and postoperative 3-dimensional head scans of 42 patients with radiologically confirmed scaphocephaly were compared to objectively measure the effect of butterfly-shaped craniectomy as performed at our institution between 2012 and 2023. The anterior and posterior as well as the overall symmetry ratio (ASR, PSR, OSR), the CR and the cranial vault asymmetry index (CVAI) were assessed at standardized levels. Furthermore, a new index defined by the ratio of frontal over posterior cranial vault volume, which is significantly reduced postoperatively is introduced presenting a feasible tool to objectify surgical results in scaphocephaly patients.

**Results:**
Mean age at surgery was 5.8 months. Pre- and postoperative head scans at the ages of 4.7 and 8.5 months were compared. Neither the ASR nor the PSR nor the OSR nor the CVAI differed significantly. Instead, at standardized levels 5 and 8 the CR was significantly increased postoperatively (69 vs. 72% at level 5, $p < 0.0001$; 68 vs. 79% at level 8, $p < 0.0001$). Additionally, the ratio of frontal over posterior cranial vault volume was reduced significantly (1.29 vs. 1.14, $p = 0.0026$), demonstrating a surgically induced shift of volume from the frontal to the posterior cranial vault.

**Conclusion:**
3-dimensional head scan is a feasible, radiation free tool to objectively measure postoperative results after surgical correction of scaphocephaly. Alongside the widely accepted CR, a new index describing the ratio of frontal over posterior cranial volume may be useful in quantifying the change of cranial vault morphology in these patients.

**Delayed treatment of scaphocephaly is associated with elevated ICP in young children**
ID: 299

**Kategorie: interdisziplinäre Chirurgie - Interdisziplinäre Chirurgie von kindlichen Fehlbildungen des Gesichts- und Gehirnschädels**

Hannah Luz[1], Peter Esslinger[1], Markus Lehner[1]

[1]*Kinderchirurgie, Kinderspital Zentralschweiz, Luzern, Schweiz*

**Background:**
Synostosis of the sagittal suture resulting in scaphocephaly is known to be the most common form of craniosynostosis. Although the severity of presentation may vary, there is a large consensus on the indication for surgical treatment due to an increased risk of intracranial pressure (ICP) and the abnormal skull shape. However, the prevalence and significance of elevated ICP remains intensely discussed for scaphocephaly patients.

**Materials and methods:**
This retrospective study focused on a cohort of five patients with delayed diagnosis of scaphocephaly, who sought medical attention at our institution between May 2022 and January 2024. Additionally, three infants diagnosed and treated for scaphocephaly during this time by osteoclastic craniectomy were used as a reference group.
The data collection involved meticulous reviews of medical records, diagnostic imaging, and clinical assessments, with a focus on demographics, clinical presentations, imaging results, CI measurements, presence of papilledema, and intraoperative documentation, including ICP measurements.

**Results:**
Over a period of two years, we analyzed five cases of patients with delayed diagnosis and treatment of scaphocephaly, with patients ranging in age from 19 to 62 months at the time of surgery. This cohort consisted of three males and two females, all displaying characteristic skull features associated with scaphocephaly.
Additionally, as a control group, we included three infants who underwent osteoclastic craniectomy within the same timeframe. These infants, two males and one female, were operated on at the ages of 8, 6, and 5 months, respectively.
Among the late treatment group, the Cranial Index (CI) was documented in four out of five cases, with a mean CI of 0.635. None of the CI values fell within the normal range. In the control group, the CI was documented in two out of three cases, with a mean CI of 0.67. There was no significant difference in the preoperative CI between the two groups ($p = 0.14$).
All patients diagnosed with scaphocephaly were assessed for papillary edema by our ophthalmologists. Two cases in the late-treatment scaphocephaly group presented with papillary edema at diagnosis, while the remaining three did not exhibit this symptom. In contrast, there were no cases of papillary edema in the control group.
All five late treatment-group scaphocephaly patients underwent modified Pi-plasty procedures with systematic intracranial pressure (ICP) monitoring. The mean opening pressure was 24mmHg, ranging from 16mmHg to 36mmHg, which significantly dropped to a mean closing pressure of 7.5mmHg.
In comparison, the control group had a significantly lower mean opening pressure of 5mmHg ($p < 0.01$).

**Conclusion:**
DeIIn conclusion, our study demonstrates that all patients in the late-treatment scaphocephaly group exhibited elevated intracranial pressure (ICP) at the time of surgery, with a mean opening pressure significantly higher than the control group. Despite variability in presentation, the elevated ICP in these patients underscores the importance of timely diagnosis and intervention to mitigate the risk of intracranial hypertension.

### Giant hairy nevus - operative strategy in facial reconstruction

ID: 657

**Kategorie: interdisziplinäre Chirurgie - Interdisziplinäre Chirurgie von kindlichen Fehlbildungen des Gesichts- und Gehirnschädels**

Klaus Exner[1]

[1]*Plastische Chirurgie Frankfurt I Hochtaunus, Oberursel, Deutschland*

**Background:**
Giant Hairy Nevus is a congenital malformation. Particularly in facial involvement the psychological trauma for the child and his family affords early surgical treatment and complete removal with optimal functional and esthetic outcome.

**Materials and methods:**
Patients with facial involvement of Giant Hairy Nevus are treated with surgical resection and reconstruction using tissue expansion, locoregional flaps and full thickness skin grafts. Surgical details of the different regions of the face have to be considered in reconstructive techniques.

**Results:**
The preoperative situation and the surgical steps of 5 children with the age from 1 – 5 yrs will be discussed.

**Conclusion:**
The optimal timetable for the surgical treatment of facial Giant Hairy Nevus is the first and second year of life. At that age children will develop less psychological trauma from hospitalization. Locoregional tissue is the first choice for reconstruction due to optimal color and texture. Superficial full thickness skin grafts are used mostly in the periorbital region.

### FAST-testing in patients with different chronic pain syndromes and spinal cord stimulation evaluation – first results of on-table testing

ID: 65

**Kategorie: interdisziplinäre Chirurgie - Operative Schmerztherapie im Interdisziplinären Kontext**

Dimitri Tkatschenko[1], Peter Vajkoczy[1], Simon Bayerl[1]

[1]*Charité – Universitätsmedizin Berlin, Klinik für Neurochirurgie, Berlin, Deutschland*

**Background:**
Spinal cord stimulation is an established therapy for patients with chronic pain where conservative treatment fails. Patients suitable for spinal cord stimulation usually undergo a trial period of several days or weeks after lead positioning and externalization of the cable extensions resulting in two surgeries. Additionally, extensions may be a source of infection. Different types of stimulations were introduced over the years with the fast-acting sub-perception therapy as the newest invention. Most current studies show significant pain reduction within 11 minutes with FAST stimulation. The aim of the current work was to evaluate the feasibility of on-table FAST-testing to predict a positive response to spinal cord stimulation.

**Materials and methods:**
All patients from May 2021 – January 2023 were identified undergoing on-table FAST testing. Pain level was documented using the NPR scale before and after 15 minutes of on-table FAST stimulation. All patients underwent the normal testing routine with cable externalization and multi-day out-of-hospital testing. The correlation of pain reduction on NPR scale and the implantation rate was evaluated.

**Results:**
23 patients were identified. In all patients FAST testing showed a decrease in NPR scale, in 21 patients the NPR scale score halved (91.3%). Mean pain reduction on NPR scale was 5.0 points. In 22 patients (95.7%) a neurostimulator was implanted after positive multi-day out-of-hospital testing.

**Conclusion:**
First experiences with on-table testing with FAST show a strong correlation with intraoperative response and response to the trial. This indicates the possibility to use intraoperative testing with FAST instead of a long and unpleasant trial period to determine the response of patients to spinal cord stimulation. This could be the first step to enable a guideline to conform all-in-one implantation after only 15 minutes of intraoperative testing.

**Portal-Venous Arterialization procedures - An overview**
ID: 845

**Kategorie: interdisziplinäre Chirurgie - Viszeralchirurgie und/oder Gefäßchirurgie bei Gefäßinfiltration in der HPB-Chirurgie**

Magdalena Menzel[1], Christopher Berlin[1], Andreas Gengenbach[1], Gabriel Stöger[1], Kim Schmid[1], Stefan Fichtner-Feigl[1], Philipp Holzner[1]

[1]*Uniklinik Freiburg - Allgemein- und Viszeralchirurgie, Freiburg im Breisgau, Deutschland*

**Background:**
Acute interruption of hepatic arterial flow can lead to fatal liver necrosis or severe biliary complications, but may also follow a subclinical course, depending on sufficient collateralization. Portal vein arterialization (PVA) is known as a salvage procedure for acute injury of the hepatic artery (HA) without possibility of arterial reconstruction, as well as for complete tumor resection involving the HA, especially in hepato-pancreato-biliary (HPB) surgery. Oxygenated blood is delivered to the liver by PVA, but hepatic inflow and portal pressure are also increased, potentially leading to portal hypertension with typical complications, e.g., refractory ascites. Experience with PVA is mainly based on case reports, case series, and some experimental animal studies. In this case series of 20 patients, different applications and techniques of PVA were gathered, with critical analysis of outcomes and complications.

**Materials and methods:**
All patients who underwent any kind of arterialization of the portal-venous system from February 2020 to February 2024 at the Department for General and Visceral Surgery, Medical Center – University of Freiburg, were included. Twenty patients in total were identified by retrospective search in the hospital's database. Follow-up data were extracted from the hospital's database; postoperative complications were assessed retrospectively using the Clavien-Dindo classification. Descriptive statistics were performed using SPSS.

**Results:**
In the collective of 20 patients, with a median age of 60.5 years, 85% underwent surgery for malignant disease, while 15% had benign disease, including two cases of chronic pancreatitis and one case of visceral artery aneurysmosis. Overall, 55% underwent pancreatic resection and 25% hepatic resection. Of the remaining 20%, one pediatric patient had recurrence of a neuroblastoma in the hepatic hilum requiring HA resection to achieve R0, one patient underwent gastrectomy with intraoperative injury of an aberrant right hepatic artery and consecutive PVA, another underwent semi-elective PVA for localized rupture of an HA aneurysm, and one patient with perihilar cholangiocarcinoma underwent resection of the bile ducts and hepatic artery without hepatic resection.
PVA was performed due to intraoperative or postoperative complications in 75% of cases, e.g., intraoperative injury or postoperative erosion of the HA, and in 25% due to other reasons, e.g., to achieve R0 resection. Fifty percent of PVAs consisted of an anastomosis between the hepatic artery and its branches with the portal vein (HA/PV), while in 30%, the splenic artery was sutured to either the portal vein or the splenic vein (SA/PV). In 20%, an artificial jump graft (JG) was used as interposition between the right common iliac artery and the portal vein.
In the postoperative follow-up, 60% (n=12) of PVA reconstructions remained open, while 15% (n=3) were thrombosed. In 25% (n=5), the PVA reconstruction was occluded or minimized interventionally due to PVA-related complications. Postoperative complications were assessed using the Clavien-Dindo Classification, with Grade 4 complications in 15% and Grade 5 in 35%. PVA-specific complications (refractory ascites and cholangitic liver abscess) were found in 45% of cases.
In search of prognostic factors for success or failure of PVA, different variables were analyzed via crosstabs and statistical analysis using the Pearson Chi-Square test. The type of PVA correlated significantly with PVA-specific complications (p=0.024), with 7 of 9 in the HA/PV group, 2 of 9 in the JG group, and zero PVA-specific complications in the SA/PV group.
No significant correlation was found between death in the postoperative course of PVA and full mobilization of the liver (p=0.658), presence of malignancy (p=0.168), complete interruption of the hepatic artery (p=0.658), type of PVA (p=0.086), type of surgery (p=0.254), or whether the PVA remained open (p=0.964).
However, resection of the bile duct (p=0.032) and whether the PVA was performed primarily or not (p=0.019) correlated significantly with death in the perioperative course. A tendency of higher risk for death was observed in patients who received PVA due to complications (p=0.058).

**Conclusion:**
Data from this collective confirms the value of PVA as a salvage procedure. The relevant mortality following PVA surgery, especially when performed secondarily in an emergent situation, underlines the importance of early decision-making and rapid execution of the procedure. In an elective setting, PVA is feasible, but complications caused by portal hypertension like refractory ascites are common and may necessitate interventional reduction of the PVA.

**Spatial Distribution and Determinants of Gastric Cancer in Urban Areas – A Spatial Ecological Study**
ID: 186

**Kategorie: interdisziplinäre Chirurgie - Viszerale Pathologie – gemeinsame Erkrankung, unterschiedliche Herangehensweise**

Stephanie Strobl[1], Roger Müller[1], Michael Kloth[1], Wilfried Roth[1], Giovenale Moirano[2]

[1]*Institut für Pathologie der Universitätsmedizin Mainz, Mainz, Deutschland*, [2]*Department of Medical Sciences, University of Turin and CPO-Piemonte, Torino, Italien*

**Background:**
Gastric cancer is one of the leading causes of cancer-related deaths worldwide, exhibiting significant geographical variability (Polk et al.). This heterogeneity can partly be attributed to different patterns of cigarette consumption, dietary habits, and especially varying rates of H. pylori infection across populations (Etemadi et al.). However, there have been no spatial ecological studies to evaluate these relationships within a geographical context yet.
Our study aimed to map the spatial distribution and determinants of gastric cancer in an urban area, focusing on the city of Mainz. The first objective was to visualize and statistically analyze the spatial distribution of gastric cancer and its known contributing factors, with an emphasis on H. pylori status and socioeconomic status (SES). The second objective was to use these findings to explore the opportunities and limitations of spatial statistical models for analyzing cancer diseases.

**Materials and methods:**
The data used in this analysis were obtained by extracting all records of gastric cancer patients with disease onset between 2019 and 2022 as reported to the local cancer registry (Krebsregister RLP). Additionally, reports of patients who received stomach biopsies were text-mined for their H. pylori status as diagnosed at the Institute of Pathology, University Medical Center Mainz. These patients were geographically mapped using the GPS coordinates of their residential addresses. The distribution of gastric cancer patients and its known determinants, such as H. pylori status and SES proxies, were then analyzed using spatial statistical models.

**Results:**
Spatial statistical models for analyzing the distribution and determinants of gastric cancer could not replicate the influence of variables known to be vital in the carcinogenesis of gastric cancer, such as H. pylori status and SES, likely due to the small number of cases (n=168). However, there was evidence of spatial clustering, indicating the presence of spatial determinants not yet captured by the model. The distribution of H. pylori positive gastric biopsies within Mainz was well-represented by spatial statistical models, likely due to the larger number of cases and the typically non-multifactorial nature of H. pylori infections compared to the process of carcinogenesis of gastric cancer. In the urban context, foreigners and individuals with a migratory background were particularly at risk for H. pylori infection, even after controlling for SES, suggesting a role for genetic susceptibility.

**Conclusion:**
Spatial statistical models are crucial tools for analyzing the spatial relationships between cancer outcomes, population attributes, and social determinants of health. Their primary contribution lies in providing a (geo)graphical overview of the spatial distribution of diseases and their underlying factors. However, for statistical evaluation, these models are limited by the small number of cases and the multifactorial nature of carcinogenesis. Nonetheless, modeling diseases and their known determinants offers valuable insights into best practices for using and further developing spatial statistical models.

**Liver Malignancies and Their Determinants in Urban Areas: A Spatial Ecological Study**
ID: 196

**Kategorie: interdisziplinäre Chirurgie - Viszerale Pathologie – gemeinsame Erkrankung, unterschiedliche Herangehensweise**

Stephanie Strobl[1], Roger Müller[1], Beate K. Straub[1], Wilfried Roth[1], Giovenale Moirano[2]

[1]*Institut für Pathologie der Universitätsmedizin Mainz, Mainz, Deutschland*, [2]*Department of Medical Sciences, University of Turin and CPO-Piemonte, Torino, Italien*

**Background:**
Although liver cancer is relatively rare in Germany, it remains one of the leading causes of cancer-related deaths due to its poor prognosis (ZfKD). Well-known individual risk factors for liver malignancies, such as liver cirrhosis, hepatitis virus infections, alcohol consumption, obesity, metabolic disorders, and congenital conditions, play a critical role in carcinogenesis (ZfKD). However, no spatial ecological study has yet explored environmental and spatial determinants influencing liver cancer. This study aims to analyze the spatial distribution and determinants of liver cancer in an urban area, offering insights into potential environmental influences.

**Materials and methods:**
Data for this analysis were obtained from records of liver cancer patients diagnosed between 2019 and 2023 at the Institute of Pathology, University Medical Center Mainz, and reported to the local cancer registry (Krebsregister RLP). These patients were geocoded based on their residential addresses and assigned to respective districts within the city of Mainz for aggregated data analysis. Spatial statistical models were used to analyze the distribution of liver cancer patients, incorporating variables such as proxies for socioeconomic status (SES), environmental factors, and social context data provided by the local statistical bureau.

**Results:**
Between 2019 and 2023, 60 liver cancer cases were diagnosed in Mainz, with a predominance of male patients (73.3%) and a mean age of 69.9 years. The most common types of liver malignancies were hepatocellular carcinoma (66.7%) and cholangiocarcinoma (16.7%), consistent with national liver cancer data. Spatial analyses found no evidence of spatial clustering. Among the environmental and spatial variables analyzed, only the proportion of female patients per district was statistically significant in explaining the spatial distribution of liver cancer cases.

**Conclusion:**
This study demonstrates that data from routine pathological diagnostics can be effectively utilized to investigate the spatial distribution and determinants of liver cancer in an urban setting. It confirmed known factors influencing liver cancer carcinogenesis, such as patient sex. Interestingly, socioeconomic status did not appear to affect the distribution of liver cancer. A key limitation of this study is the absence of data on crucial factors like chronic liver conditions and dietary habits. However, future developments in text mining and automated data extraction tools may help overcome these limitations, particularly as chronic liver conditions are also diagnosed at pathology institutes. This will provide a more comprehensive understanding of liver cancer distribution.

### Upside down - Diverticulitis in the left upper quadrant

ID: 805

**Kategorie: interdisziplinäre Chirurgie - Viszerale Pathologie – gemeinsame Erkrankung, unterschiedliche Herangehensweise**

Marie-Helen Winter[1], Fabian Scheuerlen[2], Stefan Grüne[2], Christoph Justinger[1]

[1]*Marienhaus Klinikum Hetzelstift, Klinik für Allgemein- und Viszeralchirurgie, Proktologie, Neustadt an der Weinstraße, Deutschland*, [2]*Marienhaus Klinikum Hetzelstift, Klinik für Innere Medizin 2 - Schwerpunkt für Gastroenterologie, Onkologie, Infektiologie, Pneumologie und Endokrinologie, Neustadt an der Weinstraße, Deutschland*

**Background:**
Diverticula are a common disease with increasing prevalence (1). Diverticula are predominantly located in the left hemicolon in the population of the western world. Hence patients affected by diverticulitis present typically with left sided lower abdominal pain (1). We present a rare location with atypical symptoms for a diverticulitis in the context of a case discussion.

**Materials and methods:**
Patient's characteristics:
43 year old, male patient with recurring, colicky epigastric pain for 10 years
Anamnestically the patient was status post bowel resection during childhood due to ileus. The extent of the resection remained unclear as patient and relatives were unable to provide more detailed information. Previous reports were not available. Three years before consultation a conventional cholecystectomy was performed due to cholecystolithiasis without improvement of the symptoms.

**Results:**
In the colonoscopy a colon blind sack with bland diverticula was found at an ileocolonostomy. The correlation with the computed tomography revealed indicators of a diverticulitis on the colon blind sack. Due to the level of suffering the patient desired a definitive treatment. In the explorative laparotomy the colon blind sack was located dorsal of the pancreas and reached up to the hiatus. After mobilizing the stomach, the tail of the pancreas and the spleen the colon blind sack was resected using a stapler. Histological the colon blind sack presented a chronic scarring diverticulitis.

**Conclusion:**
In the literature small intestine blind sacks at side-to-side and side-to-end anastomosis (2, 3) and colon blind sacks as a result of intestinal bypasses (4) are described. In our case a colon blind sack was found at an end-to-side anastomosis. The close anatomical location of the blind sack to the hiatus could have provided a possible explanation for the epigastric pain. In addition, diverticula were found on the colon blind sack. The histologically proven chronic diverticulitis is consistent with the for years reoccurring symptoms.

*(1) Leifeld L, Germer CT, Bohm S, Dumoulin FL, Frieling T, Kreis M, et al. S3-Leitlinie: Divertikelkrankheit/Divertikulitis. AWMF-Registernummer 021 - 020; 2022.*
*(2) Killingback M. Blind Pouch Syndrome After Bowel Resection. In: Killingback M, editor. Colorectal Surgery: Living Pathology in the Operating Room. New York, NY: Springer New York; 2006. p. 12-3.*
*(3) Killingback M. Blind Pouch Syndrome After Ileorectal Anastomosis. In: Killingback M, editor. Colorectal Surgery: Living Pathology in the Operating Room. New York, NY: Springer New York; 2006. p. 14-5.*
*(4) Ecker K, Schmidt T, Omlor G, Seitz G. Der Dickdarm-Blindsack-Bypassenteropathie oder Diversionskolitis? Zeitschrift für Gastroenterologie. 1993;31(3):205-9.*

### Artificial intelligence (AI) based sonographic differentiation between skull fractures versus sutures in small children with head injuries: A proof of concept

ID: 298

**Kategorie: SKT - Besonderheiten bei der Versorgung von Kleinkindern**

Saskia Hankel[1 1], Gerolf Schweintzger[2], Elena Friehs[1], Hesham Elsayed[1], Christof Kraxner[2], Georg Singer[1], Tristan Till[3], Sebastian Tschauner[4], Holger Till[1]

[1]*Medical University of Graz, Department of Paediatric and Adolescent Surgery, Graz, Österreich*, [2]*Hospital Hochsteiermark, Leoben, Austria, Department of Pediatrics, Leoben, Österreich*, [3]*Nanyang Technological University NTU, College of Computing and Data Science (CCDS),, Singapore, Singapore*, [4]*Medical University of Graz, Graz, Austria, Division of Pediatric Radiology, Department of Radiology, Graz, Österreich*

**Background:**
Artificial intelligence (AI) and in particular computer vision (CV) solutions could significantly enhance diagnostic accuracy and clinical decision-making in pediatric surgery. Especially in infants with head injuries the sonographic differentiation between skull fracture and normal growth plate (suture) requires pediatric radiological excellence. Since such expertise may not always be available at anytime and anywhere, this diagnostic investigation may benefit from AI based support in the future. However, such an AI solution has not be developed yet. We present the first image classification algorithm trained on sonographic images to differentiate between skull fractures and normal sutures in small children with skull injuries.

**Materials and methods:**
In this multi-institutional retro- and prospective study, 92 patients (mean age 0.78 ± 0.76 years) who underwent sonographic evaluation for suspected skull injuries were included. Images depicting a discontinuity of the skull were included. The definite diagnosis was made by pediatric radiologists. 41 of the patients sustained a skull fracture. A total of 417 images were anonymized, annotated and divided into training and validation (n=317), and testing (n=100) datasets. An EfficientNet-based convolutional neural network (CNN) was employed (variants B0 to B7).

**Results:**
The AI model demonstrated satisfactory performance. F1 score ranged from 0.82-0.92, precision ranged from 0.75-0.88 and recall ranged from 0.90-0.98 indicating adequate ability in distinguishing subtle features between fractures and sutures.

**Conclusion:**
This study introduces the first sonographic AI/ML algorithm differentiating between skull fractures and normal anatomical sutures in children. Our proof of concept lays the groundwork for further exploration of larger datasets with the potential to improve diagnostic accuracy and decision support in pediatric trauma care.

Figure 1: Grad-CAM saliency map of a skull fracture enhanced for Inference Level Visualization Technique for Deep Convolutional Neural Network Models

2D
62%
K 60
M Mittel
Aufl
2.5

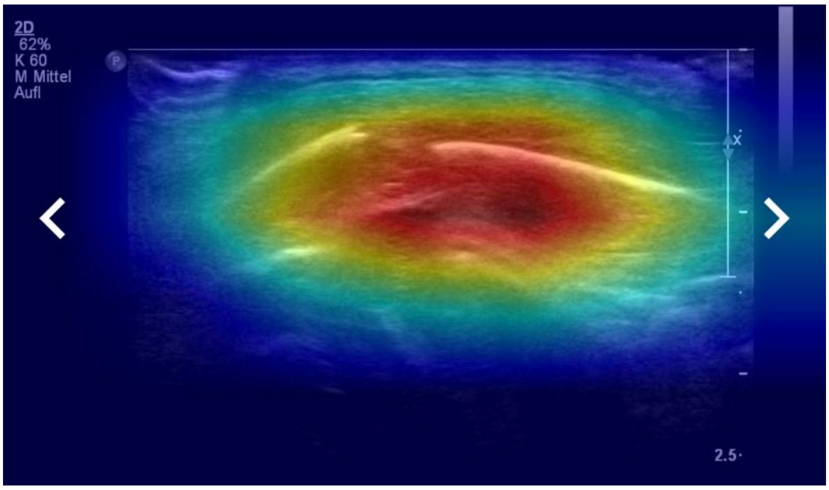

**Treatment and long-term outcomes of pediatric isolated femoral shaft fractures in the <5 year age group - results of a prospective, international, multicenter observational study**
ID: 573

**Kategorie: SKT - Besonderheiten bei der Versorgung von Kleinkindern**

Dirk Sommerfeldt[1], Claus-Uwe Weitzer[2], Christoph Aufdenblatten[3], Miriam Adrian[4], Francisco Fernandez[5], Klaus Dresing[6], Philipp Schwerk[7], Mechtild Sinnig[8], Benjamin Shore[9], Sasha Carsen[10], Kishore Mulpuri[11], Ron El-Hawary[12], Dimitri Hauri[13], Justus Lieber[14], Alexander Joeris[13], Unni Narayanan[15], Peter Schmittenbecher[16]

[1]*Altonaer Kinderkrankenhaus gGmbH, Kinder- und Jugendtraumatologie, Hamburg, Deutschland*, [2]*LKH Univ.-Klinikum Graz, Klinik für Kinderchirurgie, Graz, Österreich*, [3]*Universitäts-Kinderspital Zürich, Kinderorthopädie, Zürich, Schweiz*, [4]*Universitätsklinikum Mannheim, Klinik für Kinder- und Jugendchirurgie, Mannheim, Deutschland*, [5]*Olgahospital Stuttgart, Klinik für Orthopädie, Stuttgart, Deutschland*, [6]*Universitätsmedizin Göttingen, Klinik für Unfallchirurgie, Orthopädie, und Plastische Chirurgie, Göttingen, Deutschland*, [7]*Universitätsklinikum Dresden, Klinik und Poliklinik für Kinderchirurgie, Dresden, Deutschland*, [8]*Kinder- und Jugendkrankenhaus auf der Bult, Kinderchirurgie und -urologie, Hannover, Deutschland*, [9]*Boston Children's Hospital, Boston, Vereinigte Staaten*, [10]*Children's Hospital of Eastern Ontario (CHEO), Ottawa, Kanada*, [11]*BC Children's Hospital, Vancouver, Kanada*, [12]*IWK Health Centre, Halifax, Kanada*, [13]*AO Innovation Translation Center, Dübendorf, Schweiz*, [14]*Universitätsklinikum Tübingen, Abteilung für Kinderchirurgie und Kinderurologie, Tübingen, Deutschland*, [15]*The Hospital for Sick Children, Toronto, Kanada*, [16]*Städt. Klinikum Karlsruhe, Klinik für Kinderchirurgie, Karlsruhe, Deutschland*

**Background:**
The level of evidence for comparing treatments for femoral shaft fractures in growing children is low. This is particularly true for the age group <5 years. We present the results of a prospective, international, multicenter observational study designed to systematically collect and analyze data on treatment methods (conservative vs. surgical) and outcomes (clinical, radiological, and PROs (PROOF-LE parent scores))

**Materials and methods:**
Injury epidemiology, treatment, and follow-up data were collected from children < 16 years of age with isolated femoral shaft fractures (32-D) and open distal growth plates at European (EUR) and North American (NA) pediatric trauma centers. For age-specific analysis, only data from patients aged 0-4 years were included. Treatment was performed according to local standards; the observational study had no influence on treatment or follow-up.

**Results:**
From 03/2018 to 10/2019, 316 patients in 11 EU and 5 NA clinics were included in the study. Of these, 193 children were <5 years old: gender distribution was male:female = 116:38, side distribution was left:right = 111:82, accident severity was low:high energy = 179:14, fracture types were simple(32-D4.1 and 5.1):complex(32-D4.2 and 5.2) = 156:36. Treatment modalities included analgesia (1), spica cast (103), traction (25), combined spica/traction (13), ESIN (48), and external fixation (3). The nonsurgical:surgical ratio was 142:51. Complete follow-up (12 months) was documented in 135 of 193 patients. Complications (AE) were more frequent after surgical than after nonsurgical treatment (8/51 [15.7%, 95% CI 7.0-28.6] vs. 6/142 [4.2%, 95% CI 1.6-9.0]). An overlap of 95% CI indicates that the difference is not statistically significant. AEs in the ESIN treated patients was mainly secondary dislocation of the ESIN mandating a surgical revision. The mean (SD) time to return to full activity was 8.2 (4.2) weeks in the spica cast/traction group and 9.8 (4.7) weeks in the ESIN group, P = 0.005. The mean (SD) time to full weight-bearing was 6.2 (3.4) weeks in the spica cast/traction group and 6.5 (2.4) weeks in the ESIN group, P = 0.182. The PROOF-LE-parent scores increased continuously, reaching a mean of between 99.3 (2.4) and 98.4 (2.7) points in nonsurgically and surgically treated patients after 1 year while a statistically significant difference in PROOF-LE-parents outcome was observed (P = 0.001).

**Conclusion:**
The study makes an important contribution to the evidence base regarding epidemiology, treatment, and long-term outcomes of isolated femoral shaft fractures in the age group below 5 years. Nonsurgical treatment tends to show better results in terms of return to activity and weight bearing. However, parental observation is subjective, and no children <1 year of age were referred for surgical treatment. ESIN had led to more, but statistically not significantly so, serious adverse events than the nonsurgical treatments.

When ESIN was applied correctly, final outcomes in the surgical group were comparable to those in the nonsurgical group. This suggests that when the clinician has a thorough knowledge of all treatment options, an individualized therapy can be determined in consultation with the parents, leading to an equally good outcome.

### Case Report - Congenital postero-medial bowing of the tibia (CPMBT) in an 5 weeks old infant: a non-surgical therapeutic approach

ID: 774

**Kategorie: SKT - Besonderheiten bei der Versorgung von Kleinkindern**

Nicolas Pommer[1], Katja Schmidhofer[1], Simone Senica[1], Gregor Lettner[1], Johannes Schalamon[1], Christoph Arneitz[1], Bernhard Kienesberger[1]

[1]*Klinikum Klagenfurt am Wörthersee, Kinder- und Jugendchirurgie, Klagenfurt, Österreich*

**Background:**
Tibia recurvata congenita, or congenital posteromedial bowing of the tibia (CPMBT), is a rare condition involving an isolated deformity of the lower leg, where the tibia and fibula are bent in two planes, resulting in a valgus deformity and anterior foot bending. This unilateral malformation typically occurs without associated anomalies, and its incidence is unclear due to limited data. While amniotic bands or fetal malposition are suspected causes, the exact etiology remains unknown.
Although many authors suggest CPMBT may self-correct, data on untreated outcomes is scarce. The condition is known to cause limb length discrepancies, often necessitating lengthening surgery. Conservative treatment outcomes are underreported, underscoring the relevance of this case. To the best of our knowledge, no previous literature has documented conservative treatment for this condition.

**Materials and methods:**
This case report describes the conservative treatment of Tibia recurvata congenita. We present the case of a male infant born at term in March 2024 with posteromedial bowing of the left tibia and fibula. At birth, the valgus deformity measured approximately 30°, with posterior recurvation of about 60°.
At 6 weeks of age, the first therapeutic intervention was performed in the operating room under sedoanalgesia with spontaneous breathing. A controlled tibial fracture was carefully induced by gradually bending the lower leg, achieving partial correction of the recurvation. A corrective thigh cast was then applied, initially reducing the tibial axis by 30°. One week later, the cast was replaced in the OR, and the principle of distraction osteogenesis was employed for an additional 4 weeks.

**Results:**
The short- term- outcome for the conservative treatment of the patient is satisfactory.
Six weeks after the initial procedure, clinical evaluation showed favourable results, with normal ankle and knee mobility. Radiographs revealed a reduction in tibial recurvation to 22° and medial bowing to 14°. Subsequent clinical follow-ups indicated satisfactory growth and axis development of the affected leg. However, there remains a slight shortening and internal rotation of the left lower limb.
Currently, treatment consists of wearing a lower leg orthopaedic splint throughout the day. A follow-up evaluation is scheduled in 4 months, with the expectation of observing continued growth and improved alignment of the leg axis.

**Conclusion:**
CPMBT is a rare congenital condition, and as a result, no evidence-based clinical or treatment guidelines currently exist. This often leads to a delayed need for lengthening surgery. This case report demonstrates the potential for favorable short-term outcomes using non-invasive conservative cast treatment for this congenital deformity of the lower leg. In future clinical follow-ups, we anticipate further alignment of the tibial axis and a reduction in limb length discrepancy. We hope that this documented treatment approach contributes to a better understanding of the disease and ultimately facilitates the development of effective therapeutic options.

### Limb salvage after severe trauma of the foot with double lap technique - a case report of an 8-year-old boy after lawnmower accident

ID: 431

**Kategorie: SKT - Gelenkverletzungen und Gelenkinstabilitäten**

Ina Fichtel[1], Holger Bannasch[2], Joerg Bayer[3]

[1]*Schwarzwald-Baar-Klinikum, Unfallchirurgie und Orthopädie, Kinderorthopädie, Villingen-Schwenningen, Deutschland*, [2]*Schwarzwald-Baar-Klinikum, Plastische-, Hand- und Ästhetische Chirurgie, Villingen-Schwenningen, Deutschland*, [3]*Schwarzwald-Baar-Klinikum, Villingen-Schwenningen, Deutschland*

**Background:**

Amputation injuries in children commonly result from accidents involving motor vehicles, agricultural machinery, and notably, lawn mowers. The management of injuries related to lawn mowers is particularly challenging due to significant wound contamination, the presence of avascular tissue, soft tissue deficits and exposed bone. A variety of treatment options are available, and often, a sufficient supply of prosthetics is essential for effective rehabilitation. We report on an 8-year-old boy who got under a ride-on lawn mower and sustained 3$^{rd}$ degree open fracture of the calcaneus, 3$^{rd}$ degree open fracture of the tarsal bone and 3$^{rd}$ degree open fracture of the metatarsal bone 3-5. The 5$^{th}$ toe was amputated in the metatarsophalangeal joint. There was a severe soft tissue injury with significant wound contamination, subtotal transection of the Achilles tendon and exposed proximal portion of the gastrocnemius muscle. Although amputation was considered during the initial procedure, we opted for radical debridement and stabilization of the calcaneus and cuboid fractures as well as reconstruction of the Achilles tendon. For further procedures we developed a careful planning with different therapeutic approaches to achieve full-weightbearing and normal gait pattern.

**Materials and methods:**

After radical debridement of the soft tissue the calcaneus and metatarsal fractures were reduced and stabilized with K-wires. The Lisfranc joint was transfixed, and a modular external fixator was applicated. The tissue was regularly and consistently debrided, and necrotic tissue was removed. Finally, the heel and calf were covered with a latissimus dorsi flap, while the dorsum of the foot was covered with a parascapular flap. Split-skin grafting was performed in subsequent procedures.

**Results:**

Our patient was discharged on day 60. A fluid gait with crutches was possible 3 months after the injury. The flap reconstruction is well vascularized, and the sole of the foot has full sensitivity. The shape of the foot has been restored in such a way, that wearing a shoe with appropriate insole support is possible.

**Conclusion:**

By employing our treatment algorithm with immediate fracture fixation and early plastic reconstruction, we aimed to avoid bone infection and maintain the shape of the foot to enable the participation in regular physical activities and hopefully preserve bone growth.

**analysis of growth disturbances after surgical treatment of apo- and epiphyseal fractures of the pediatric knee joint**
ID: 627

**Kategorie: SKT - Gelenkverletzungen und Gelenkinstabilitäten**

Nina Meyerhofer[1], Markus Geßlein[2], Hermann Josef Bail[2]

[1]*Klinikum Nürnberg, Campus Süd / Südklinikum, Abteilung für Kinderchirurgie und - urologie, Nürnberg, Deutschland*, [2]*Klinikum Nürnberg, Campus Süd / Südklinikum, Abteilung für Unfallcirurgie und Orthopädie, Nürnberg, Deutschland*

**Background:**
Fractures of the infantile knee joint are rare. If these injuries affect the growth plate, they can lead to long-term consequences, such as growth disorders that manifest as leg length discrepancy, which can limit the functionality of the knee joint. This study aims to determine the relationship between the occurrence of complications and possible predisposing factors

**Materials and methods:**
The data basis included 31 patients younger than 18 years with a diagnosis of fracture with apo- or epiphyseal involvement of the distal femur or proximal tibia. The data collected were from the patients' digital medical records, operative reports, radiographs and findings. The Kruskal-Wallis test, as well as the Fisher's Exact test, were used to identify possible risk or prognostic factors.

**Results:**
The mean age was 13.3 years (SD ± 4.5 years). 28 (90.7%) of our patients were male, two were female (9.7%). Proximal tibial fractures (64.5%) were more common than distal femoral fractures (45.5%). The ratio of affected extremity was 2:1 for left to right. Fractures of the Salter-Harris classification were two times more frequent than those of the Watson-Jones type. The most common accident mechanisms were traffic accidents and sports accidents while playing soccer. Open reduction with internal fixation (ORIF) was the most commonly used treatment method. The functionality of the injured knee joints was recorded using the IKDC score, among others, which averaged 97% (SD ± 8.8%). The complication rate was 38.7%, of which 19.4% were relevant leg length discrepancies (>1.5cm). Distal femur fractures of the Salter-Harris type are associated with the highest complication rate. A period longer than five days between injury and surgery favors the development of leg length discrepancies.

**Conclusion:**
Early surgical treatment by anatomic reduction and stable fixation is therefore mandatory to prevent growth disturbances.

**Surgical treatment, complications and clinical-radiological outcome of fractures close to the joint with resorbable screws.**
ID: 828

**Kategorie: SKT - Gelenkverletzungen und Gelenkinstabilitäten**

Tobias Jhala[1], Markus Dietzel[1], Simon Scherer[1], Justus Lieber[1], Jörg Fuchs[1]

[1]*Universitätsklinikum Tübingen Abteilung für Kinderchirurgie mit Poliklinik, Tübingen, Deutschland*

**Background:**
Resorbable screws are increasingly being used in traumatology. The non-necessary removal of osteosynthesis material is particularly attractive. In children in particular, the use of absorbable screws can lead to an improvement in patient care and cost, as a second procedure to remove osteosynthesis material under general anaesthesia is no longer necessary. The ideal composition of the screws to ensure stable osteosynthesis beyond fracture healing and to avoid premature resorption / material breakage and thus fracture dislocation is currently unclear.

**Materials and methods:**
All fractures treated with resorbable screws in the period 2020 - 2023 were evaluated and compared with a group of patients with similar fractures treated in the same period and stabilised with conventional screws.

**Results:**
A total of 9 patients (seven radial condyle avulsion fractures, two distal tibia fractures) were treated with absorbable screws over a period of three years. The treated radial condyle avulsion fractures were compared with a group treated with conventional screws over the same period. The functional outcome was restitutio ad integrum in all patients, but in the group of patients treated with resorbable screws, 2 screw bridges occurred shortly after implantation. As there was no secondary dislocation, no revision surgery was necessary. A resorbable screw inserted in the OSG was seen as a possible cause of a developed CRPS and was revised and re-drilled before resorption. The group of patients treated with absorbable screws underwent significantly more frequent radiological monitoring until consolidation.

**Conclusion:**
Surgical treatment with absorbable screws in children is a promising option for avoiding secondary surgery (removal of osteosynthesis material), but must be weighed against the risk of material fracture and more frequent radiological checks. The risk of material breakage can be minimised by improving the screw composition. The more frequent use of radiological follow-up checks will also decrease once improved screws have been established.

### Traumatic Chylothorax – A Case Report

ID: 836

**Kategorie: SKT - Stammverletzungen**

Maximilian Holweg[1], Markus Dietzel[1], Christoph Slavetinsky[1], Justus Lieber[1], Jörg Fuchs[1]

[1]*Universitätsklinikum Tübingen, Kinderchirurgie und Kinderurologie, Tübingen, Deutschland*

**Background:**
Traumatic chylothorax is a rare condition characterized by accumulation of chyle in the pleural cavity. The pleural effusion is typically resulting from injury to the thoracic duct or the lymphatic system during trauma. Trauma, whether due to blunt force, penetrating injuries, or most common surgical interventions, can disrupt the normal flow of lymphatic fluid, leading to leakage into the pleural space. The management of chylothorax often involves a combination of dietary modification, such as initiating a low-fat or fat-free diet, drainage of the pleural effusion, and in some cases, surgical interventions.

**Materials and methods:**
Case Report: We present a case of a two-year-old with traumatic chylothorax after falling from height. The patient was admitted to our resuscitation room of our level I trauma center after falling from a height of approximately five meters. The initial primary and secondary survey was unremarkable. Focused Assessment with Sonography for Trauma showed no intraabdominal injury or pleural effusion, chest X-ray showed no abnormalities. After an uneventful surveillance on the pediatric intensive care unit, the patient was admitted to the pediatric surgical ward. One day after admission, the patient complained of pain in the thoracic spine followed by an X-ray with the incidental finding of a giant pleural effusion with mediastinal shift in the left hemithorax without any respiratory symptoms. CT scan showed pleural effusions, larger on the left than the right, with density values consistent with an exudate, without active bleeding and no indication of hemothorax. A chest drain was placed on the left hemithorax, fractionally draining chylous fluid with elevated triglycerides (1263 mg/dl). Conservative management including fat free food was initiated. On day two, a peripherally inserted central venous catheter (PICC line) was placed to initiate parenteral nutrition, and the patient was kept NPO, except for water. Under these measures, the drainage produced progressively less fluid and could be removed without complications on the eighth day. The patient was discharged after eleven days in hospital without pleural effusion, with full parenteral nutrition via PICC line. After four weeks, the patient was readmitted to initiate a fat-free diet, followed by regular diet. There was no pleural effusion in sonography and was discharged after three days.

**Results:**
Chest trauma is the second leading cause of death in pediatric trauma. Chylothorax occurs when the thoracic duct is injured or obstructed, causing chyle to drain into one or both pleural cavities. The duct crosses from right to left at the level of the fifth thoracic vertebra, and the location of the injury determines which side is affected. In our case, the pleural effusion was on the left side. Chylothorax often develops gradually, typically within two to ten days after trauma, though it can take up to 25 days, with an average onset of seven days. In our case, there was no pleural effusion at admission and the patient developed a pleural effusion at day one. Management includes conservative, minimal invasive and surgical treatment options. Conservative treatment includes a stepwise approach and starts with the insertion of a chest tube or thoracocentesis, followed by dietary modifications (fat free diet, medium-chain triglyceride diet, total parenteral nutrition), somatostatin (Octreotide) to decrease chyle flow and pleurodesis. Surgical treatment options are ligation of thoracic duct, pleurodesis and pleuroperitoneal shunts. In this case, the patient received a fat free diet followed by total parenteral nutrition for four weeks as a stepwise conservative approach without further escalation. We were able to remove the drain after eight days and the patient was discharged with total parenteral nutrition. The outcome in our patient was good without severe morbidity.

**Conclusion:**
Traumatic chylothorax is an uncommon complication of blunt chest trauma that may take several days to manifest as a pleural effusion. Due to this delayed onset, it's crucial to monitor for late complications in children with blunt chest injuries.

**Introducing the Forearm Fracture Index to Define the Diametaphyseal Junction Zone through Clinical Evaluation in a Cohort of 366 Diametaphyseal Radius Fractures**
ID: 87

**Kategorie: SKT - Varia**

Christoph von Schrottenberg[1], Ricardo Beck[1], Susann Marie Beck[1], Eric Waltersbacher[1], Christian Kruppa[1], Alexandra Wilke[1], Matthias Kuhn[2], Philipp Schwerk[1], Guido Fitze[1], Jurek Schultz[1]

[1]*Universitätsklinikum Carl Gustav Carus Dresden, Klinik und Poliklinik für Kinderchirurgie, Dresden, Deutschland*, [2]*Medizinische Fakultät Carl Gustav Carus, Institut für Medizinische Informatik und Biometrie, Dresden, Deutschland*

**Background:**
Unstable diametaphyseal radius fractures (DMRFs) can be prone to complications, and treatment strategies are heterogeneous. Studies are difficult to interpret as definitions of the diametaphyseal junction zone (DMJZ) are impractical for clinical use, imprecise, or prone to error.

**Materials and methods:**
We introduce the forearm fracture index (FFI) to define DMRFs in radiographs and ultrasound. The FFI is calculated by the ratio of the fracture's distance to the distal radius growth plate over the width of the radius growth plate. The higher the FFI, the more proximal the fracture is. We define DMRFs to have an FFI between 1 and 2. All DMRFs treated at our institution between 2010 – 2020 were identified, and demographic data, fracture characteristics, and therapeutic strategies were assessed retrospectively. Comparative sub-analysis was performed between DMRFs(-) as defined in previous publications (Lieber & Sommerfeldt, 2011) and DMRFs(+) that were more proximal but still met our criteria.

**Results:**
516 DMRFs were identified, representing 13.0% of all screened radius fractures. Excluding buckle fractures and patients lost to follow-up, 366 DMRFs were eligible for further analysis. Conservatively managed DMRFs were more distal than those managed operatively, represented by a lower FFI (1.28 vs. 1.34, $p = 0.0051$). 21 (5.7%) of all DMRFs were identified as DMRFs(+). These were significantly more dislocated and necessitated surgery more often than DMRFs(-) (52.4 vs. 24.6%, $p = 0.009$).

**Conclusion:**
The FFI may be a good tool to identify and describe DMRFs. It can help guiding treatment decisions and make future studies on this entity more comparable.

**Enhancing Telemedicine Communication for Improved Outpatient Pediatric Trauma Care**
ID: 202

**Kategorie: SKT - Varia**

Nariman Mokhaberi[1], André Strahl[2], Benjamin Schoof[1 2], Konrad Reinshagen[1], Kristofer Wintges[1]

[1]*Universitätsklinikum Hamburg-Eppendorf, Klinik und Poliklinik für Kinderchirurgie, Hamburg, Deutschland*, [2]*Universitätsklinikum Hamburg-Eppendorf, Klinik und Poliklinik für Unfallchirurgie und Orthopädie, Hamburg, Deutschland*

**Background:**
Pediatric traumatology is a complex field that requires a comprehensive understanding of physeal development, remodeling potential, and the ossification process in order to ensure appropriate patient treatment. Although there have been numerous promising approaches to implement telemedicine in pediatric trauma care, these processes have not yet been regularly established in outpatient pediatric traumatology due to barriers faced by both physicians and patients. Patients reported several concerns, including misconceptions and fear, as well as technical incompetence and the potential need to use new devices. Physicians, for their part, cited a number of issues, including licensure issues, reimbursement for telemedicine services, and malpractice liability.
The objective of this study was to ascertain the willingness of general practitioners to participate in telemedical collaboration within the field of pediatric traumatology and to identify potential factors that may impede this willingness. Improving such cooperation could significantly enhance the collaboration between outpatient and inpatient sectors and promote digitalization in pediatric traumatology.

**Materials and methods:**
A survey consisting of 15 items was sent to 800 practicing trauma surgeons, pediatric surgeons, and pediatricians within a 100 km radius of Hamburg, Germany. The survey included questions about the respondents' professional experience and telemedicine experience, as well as inquiries about possible telemedicine collaborations.

**Results:**
The response rate was 19.3%. Less than half of the participants already used telemedicine in daily practice. In general, 75% of respondents expressed an interest in collaborating with the inpatient sector. The most common reasons for hospital referral were the need for surgery, inadequate treatment of children in practice and co-assessment. The majority were in favor of flexible communication, either via video telephony, imaging applications like or messaging applications.

**Conclusion:**
There has already been evidence that the use of telemedicine can be a supportive tool in medicine to the benefit of both physicians and patients. Within the pediatric traumatology outpatient sector, our findings revealed a substantial interest in telemedicine collaboration. Collaboration between outpatient clinics and hospitals in telemedicine can significantly reduce treatment redundancy, thereby lowering duplicate costs and unnecessary administrative burdens on health authorities. This is especially crucial given the growing scarcity of doctors and limited hospital resources [17,18]. The exchange of information should be tailored to meet individual needs, providing practitioners with a versatile and personalized approach that includes the capability for image processing. It is imperative that data protection regulations are strictly adhered to.
Further prospective research is required to evaluate the effectiveness of telemedicine collaboration in treating pediatric trauma in both hospital and outpatient settings. Ultimately, foreign healthcare systems that have similar treatment processes and comparable problems at the interface between the outpatient and inpatient sectors can adopt implemented telemedical cooperation.

**Epidemiological pattern of metaphyseal-diaphyseal junction forearm fractures in children and adolescents**
ID: 265

**Kategorie: SKT - Varia**

Markus Dietzel[1], Charlotte Moog[1], Matthias Schunn[2], Hans Joachim Kirschner[1], Jörg Fuchs[1], Justus Lieber[1]

[1]*Universitätsklinikum Tübingen, Kinderchirurgie, Tübingen, Deutschland*, [2]*Charité Universitätsmedizin, Klinik für Kinderchirurgie, Berlin, Deutschland*

**Background:**
Forearm fractures are most common fractures in children and adolescents. They are divided into various types of fractures and also judged upon location (e.g., distal, diaphyseal and proximal fractures), each of which has different levels of stability and, together with the individual correction potential, is subject to different treatment concepts. For fractures of the distal metaphyseal-diaphyseal junction (MDJ) of the forearm – which are not listed in any classification – there is no reliable data on incidence, peak age and correction potential. Therefore, there are no evaluated treatment recommendations. In a retrospective data analysis, epidemiological data on all forearm fractures in children and adolescents from a 15-year period are to be summarized. In particular, detailed evaluation is to be conducted of MDJ fractures, which are delimited by distal and diaphyseal fractures and therefore have overlaps but also special features.

**Materials and methods:**
The MDJ of the forearm was defined as the square over the joints of both forearm bones subtracted with the square over the metaphysis of the radius alone. The data of patients <16 years of age who were treated with a forearm fracture at a single high-volume pediatric trauma center were retrospectively analyzed over a 15-year time period. All cases with a fracture of the MDJ region of the forearm underwent a detailed examination focused on demographics, accident context, fracture conditions, treatment, and outcome. Study conduction was approved by the local ethical committee.

**Results:**
In the recording period from 01.01.2010 to 31.12.2019, at the author's pediatric trauma center, 2789 cases with forearm fractures at <16 years of patient age were documented using ICD coding. Due to simplified data retrieval from hospital records, forearm fractures included all fractures of the both bones radius and ulna and thus radial neck and olecranon fractures were included in overall statistics. Preliminary data showed 38% of the above-mentioned forearm fractures to be located on the distal third of the forearm (distal shaft, MDJ, metaphyseal, and epiphyseal fractures). 11% of all forearm fractures were fractures of the metaphyseal-diaphyseal junction region. These preliminary results showed most MDJ fractures to occur in children during primary school age (6-10 years) whereas overall forearm fracture statistics see a broader age distribution with a homogeneous fracture incidence from preschool through to adolescence. Gender ratio was boys–girls 1,5 : 1 in all forearm fractures and 2:1 in MDJ fractures. Falls from scooter/skateboard/in-line skates and accidents during soccer play were the leading causes of injury in MDJ fractures, whereas other low-velocity trauma were leading in none-MDJ distal forearm fractures over bicycle and sports accidents.

**Conclusion:**
The MDJ forearm fracture, as an independent entity between shaft and metaphyseal fractures, presents with its own features regarding epidemiological pattern as well as treatment requirements: According to literature, conservative treatment yields good results if the initial position and the subsequent consolidation are adequately monitored radiologically. For surgical treatment, several options with low potential for complications are available, including transepiphyseal-intramedullary K-wire and modified ESIN techniques as well as ORIF using plate osteosynthesis in older patients. Due to their special nature both from an epidemiological as well as from a mere medical point of view, MDJ fractures should be recognized as an independent entity and as such find their way into the current pediatric fracture classifications.

**Piloting of a simulation-based training in Brazilian-German partnership on presentation, diagnosis and initial management of severely traumatized pediatric patients**
ID: 494

**Kategorie: SKT - Varia**

Sinan Bakir[1 2], Alexander Hönning[3], Janna Schwab[1], Erik Haucke[4], Alexander Jünemann[4], Uwe Weibrecht[5], Fabiana Teixeira de Carvalho Portela[6], Samuel MacHado Martins[7], Rodrigo Oliveira Ribeiro[8], Ekkernkamp Axel[1], Jakob Olbrich[4]

[1]*BG Klinikum Unfallkrankenhaus Berlin, Klinik für Unfallchirurgie und Orthopädie, Berlin, Deutschland*, [2]*Universitätsmedizin Greifswald, Greifswald, Deutschland*, [3]*BG Klinikum Unfallkrankenhaus Berlin, Zentrum für klinische Forschung, Berlin, Deutschland*, [4]*BG Klinikum Unfallkrankenhaus Berlin, Zentrum für Notfalltraining, Berlin, Deutschland*, [5]*ProBrasil, Sao Raimundo Nonato, Brazil*, [6]*Universidade Estadual do Piauĺ , Teresina, Brazil*, [7]*Secretaria de Estado da Saúde do Piauí , Teresina, Brazil*, [8]*Serviço de Atendimento Móvel de Urgência, Sao Raimundo Nonato, Brazil*

**Background:**
With more than 20 deaths per 100,000 inhabitants each year, Brazil is one of the countries with the highest number of road traffic victims. The situation is particularly worrying in the federal state of Piauí in northern Brazil, where hospitals are far apart. There is no organized emergency care and a lack of special training for prehospital emergencies results in the second highest mortality rate due to traffic in the entire country. The development of an emergency training program in a Brazilian-German cooperation to train medical staff in the emergency care of pediatric patients is intended to counteract these shortcomings. The main objective of this 2-year project is to investigate whether the training program can be successfully implemented, accompanied by an improvement of health care professionals' individual skills in emergency care. Thereby, we want to achieve timely access to adequate surgical care for everyone, particularly including the vulnerable group of children.

**Materials and methods:**
In order to fully adapt the project to regional circumstances and ensure a successful and sustainable implementation of the training, an electronic survey was initially distributed to local medical staff to determine and address training needs. The concept based on this feedback was further developed with local experts and consists of theoretical and practical course units. In the theoretical part, medical staff is taught specialist knowledge about the presentation, diagnosis and initial care of pediatric patients with severe trauma, as well as a standardized trauma management protocol and the principles of crew resource management (CRM). In simulation-based training sessions, various case scenarios are practiced with the aid of resuscitation manikins.
In the pilot phase, the final concept was tested for feasibility and potential for improvement. On day one, instructors from the German project team taught the contents of the course to the Brazilian participants. On day two, the Brazilian participants themselves acted as instructors in order to be able to pass on the course content themselves in the future. The aspects to be evaluated were the content to be taught, methods of knowledge transfer, aids and materials used as well as organizational aspects. In the end of February 2024, training participants and instructors were invited to give feedback on the training course by means of an electronic survey.

**Results:**
98 healthcare professionals with an average working experience of 9.7 ± 6.27 years participated in the initial survey, of which 37 (37.8%) were nurses, 24 (24.5%) nursing technicians, 8 (8.2%) physicians, 2 (2.0%) paramedics and 26 (26.5%) had another profession. The response format was predominantly a 5-point Likert scale with the options from “strongly agree” to “strongly disagree”. While 63.9% of the medical staff considers themselves capable of treating complex trauma in adults, the proportion for treating pediatric complex trauma was only 43.5% ($\chi^2(4) = 10.4$, $p = 0.035$). The participants also feel much more confident in applying the current resuscitation guidelines when dealing with adults (91.2% strongly/somewhat agree) than with children (70.2% strongly/somewhat agree).
The participation rate in the electronic survey following the emergency training was 100% (38/38 training participants and 4/4 instructors). Materials and lectures developed were evaluated by the vast majority of instructors (75-100% strongly/somewhat agreed depending on the item) and participants (94-100% strongly/somewhat agreed) as relevant and of an appropriate standard. The case scenarios were assessed to be well chosen and relevant to practice according to 97% of the participants. All participants stated that they would recommended the course to others, and 80% stated that they had been well prepared for the course content and now felt able to carry out the training course as instructors. However, all course instructors and 50% of the training participants expressed the wish to extend the duration of the training. Moreover, 90% of the training participants wish to attend follow-up training courses to deepen both their theoretical knowledge and their presentation skills.

**Conclusion:**
The survey of 98 healthcare professionals showed that there is a need for training in the field of pediatric emergency care, especially in the treatment of complex trauma and in the knowledge of current resuscitation guidelines. In the pilot phase, the emergency training was rated as very positive across all aspects examined. Instructors' and participants' feedback was helpful to develop and improve the training concept. In the further course of the project, it is to be investigated if the emergency training can be implemented sustainably through the multipliers and whether it leads to an improvement in the patient care skills of the medical staff involved to guarantee safe trauma surgical skills for everyone.

### Double-prebent Intramedullary Nailing for Distal Diaphyseal-Metaphyseal Radius Fractures in Children: A Retrospective Study in two German Pediatric Trauma Centers

ID: 528

**Kategorie: SKT - Varia**

Jakob Warmbrunn[1], Ludger Tueshaus[2], Carsten Krohn[1]

[1]*München Klinik Schwabing, Department of Pediatric Surgery, München, Deutschland*, [2]*Universitätsklinikum Schleswig-Holstein, Campus Lübeck, Department of Pediatric Surgery, Lübeck, Deutschland*

**Background:**

The treatment of displaced distal diaphyseal-metaphyseal radius fractures in children is challenging. When osteosynthesis is necessary, options include closed reduction with percutaneous K-wire fixation, plate osteosynthesis, or mini-external fixation. Antegrade ESIN (Elastic Stable Intramedullary Nailing) or single pre-bent retrograde ESIN have also been described [1].

In 2022, a case series was published on closed reduction with retrograde double-pre-bent intramedullary nailing of the radius in these fractures. This new technique aims to achieve three-point fixation of the radius using a minimally invasive approach [2]. This study retrospectively examines the application of this technique at two German pediatric trauma centers.

**Materials and methods:**

We retrospectively analyzed cases from the Departments of Pediatric Surgery at München Klinik Schwabing and University Hospital Schleswig-Holstein between 2022 and 2024. Children (5-14 years old) with displaced distal diaphyseal-metaphyseal radius fractures treated using the described osteosynthesis method after publication of the new technique were included. Patient characteristics and surgical details were extracted from patient records. Follow-up X-rays were evaluated for displacement, material positioning, and bone healing. Documented complications and potential problems were analyzed.

**Results:**

We identified 25 children who underwent the described procedure. The average age was 9.3 years (range 4–14 years), with more boys (68 %) than girls affected. In 19 of 25 patients, the left forearm was involved. In 12 cases, surgery was performed on the day of the accident, with the latest surgery occurring on day 13. The average operation time was 39 minutes (range 15–90 minutes), with an average fluoroscopy time of 1:04 minutes and a mean dose-area product of 0.775 Gy*cm². Five patients were treated with steel nails and twenty with titanium nails. Postoperatively, all patients received a plaster splint for 2 weeks. The intraoperative and short-term (<1 week) postoperative course was complication-free. During regular follow-ups, one patient developed a pseudobursa at the radial nail entry site, which became inflamed and required early material removal. In one case, due to a multi-fragmentary fracture, the nail had integrated into the callus in such a way that minimally invasive removal was not possible. The remaining patients showed normal healing.

Nail removal occurred after radiological consolidation, on average after 4.5 months (range 2.3–9.2 months), which is longer than the usual 3 months for diaphyseal fractures. At the time of nail removal, there was an average axial deviation of 6.8° in the anteroposterior plane and 4.3° in the lateral plane. All fractures healed in good radiological alignment, with no documented functional issues.

**Conclusion:**

This retrospective study shows that double-bent ESIN is suitable for managing distal diaphyseal-metaphyseal radius fractures.

Jianyi et al. (2023) demonstrated in a small comparative study that this technique might have advantages over K-wire fixation and antegrade ESIN in terms of operation time, radiation exposure, fracture alignment, and complications [3]. We recommend delayed metal removal after 4-6 months to prevent the nail from becoming lodged in the endosteal callus. Excessive protrusion of the ESIN at the cortical entry point should be avoided.

*1. Cai H.Z., Wang Z., Cai H. Prebending of a titanium elastic intramedullary nail in the treatment of distal radius fractures in children. Int Surg 2014; 99(3): 269-75.*
*2. Krohn C. Double Pre-Bending of an Intramedullary Nail Is the Minimal Invasive Osteosynthesis Solution for Dia-Metaphyseal Fractures of the Radius in Children: Technical Note and Case Series. Children (Basel) 2022; 9(4).*
*3. Jianyi J., et al. Comparison of three fixation methods in pediatric metaphyseal-diaphysis junction fracture of the distal radius: a retrospective study in two centers. Front Pediatr 2023; 11: 1244704.*
